# PMMA-Based Wafer-Bonded Capacitive Micromachined Ultrasonic Transducer for Underwater Applications

**DOI:** 10.3390/mi10050319

**Published:** 2019-05-11

**Authors:** Mansoor Ahmad, Ayhan Bozkurt, Omid Farhanieh

**Affiliations:** 1 Faculty of Engineering and Natural Sciences, Sabanci University, 34956 Istanbul, Turkey; abozkurt@sabanciuniv.edu (A.B.); farhanieh@sabanciuniv.edu (O.F.); 2 Sabancı University Nanotechnology Research and Application Center (SUNUM), 34956 Istanbul, Turkey

**Keywords:** Capacitive Micromachined Ultrasonic Transducers (CMUTs), wafer bonding, PMMA, underwater acoustics, Microelectromechanical Systems (MEMS)

## Abstract

This article presents a new wafer-bonding fabrication technique for Capacitive Micromachined Ultrasonic Transducers (CMUTs) using polymethyl methacrylate (PMMA). The PMMA-based single-mask and single-dry-etch step-bonding device is much simpler, and reduces process steps and cost as compared to other wafer-bonding methods and sacrificial-layer processes. A low-temperature (<$180{\;}^{\circ }C$) bonding process was carried out in a purpose-built bonding tool to minimize the involvement of expensive laboratory equipment. A single-element CMUT comprising 16 cells of 2.5 mm radius and 800 nm cavity was fabricated. The center frequency of the device was set to 200 kHz for underwater communication purposes. Characterization of the device was carried out in immersion, and results were subsequently validated with data from Finite Element Analysis (FEA). Results show the feasibility of the fabricated CMUTs as receivers for underwater applications.

## 1. Introduction

Capacitive Micromachined Ultrasonic Transducers (CMUTs), a potential alternative for piezoelectric ultrasonic transducers, have been under extensive development since their introduction in the mid-1990s [1,2,3,4,5,6,7,8,9,10]. Initially developed for air-coupled applications, CMUTs have shown far better acceptability in immersion applications, including medical ultrasonic imaging, medical therapy, and underwater imaging [11,12,13,14,15].

CMUTs are parallel-plate capacitors fabricated using Microelectromechanical System (MEMS) technology [16,17]. The sacrificial-release and wafer-bonding processes are the two methods used in the production of CMUTs [2,18]. The sacrificial-release-process technique is a surface micromachining process where a sacrificial layer is patterned on a substrate that defines the shape, size, and cavity height of CMUTs. A membrane is deposited on the top of the sacrificial layer, and then the sacrificial layer is released via etch holes with either wet or dry etching. A number of adjustments, optimizations, and selection issues are associated with sacrificial-release-process-based CMUTs, such as alignment, inclusion of etch holes into the design, and selection of material for the sacrificial layer, membrane, and etchants [19]. Furthermore, problems associated with sacrificial-layer underetching are the lack of control over underetching. Hence, cavity-height and membrane-stiction issues arise. For low-frequency operations, a relatively large membrane radius and controlled cavity height are required. However, in large-size devices, it is difficult to sustain a suspended membrane using the underetching method. Therefore, the wafer-bonding method was adopted to overcome the membrane-collapse issues. The wafer-bonding process is a surface micromachining technique that does not involve the sacrificial-release process. This process requires a prime wafer and an silicon-on-insulator (SOI) wafer; the cavity is defined on the prime wafer during patterning and bonded to the SOI wafer. The handle and buried-oxide layers of the SOI wafer are then removed, leaving the silicon-device layer, which acts as the membrane [17]. Silicon nitride-deposited wafers can also be used as an alternative to the SOI wafer. The silicon nitride-deposited wafer is bonded to the substrate, the silicon layer is then etched, and the nitride layer forms the membrane [20]. The wafer-bonding process ensures very controlled cavity height and uniform membrane thickness, which is defined by the device layer of the SOI wafer or the silicon nitride wafer [21,22].

Anodic bonding, metal bonding, adhesive polymers, and wafer direct bonding are the typical bonding methods used for CMUT fabrication [5,6,22,23,24,25,26]. Wafer bonding encounters yield issues due to the roughness of the wafers’ surface and contamination at the bonding surfaces [22]. The problem is addressed by using SOI wafers, but producing suitable SOI wafers is very costly and complex [18,19]. To avoid SOI wafers, a silicon nitride-deposited wafer can also be bonded to the substrate. The silicon layer is removed in the subsequent steps, and the nitride layer acts as the membrane [20]. Another issue associated with wafer-bonding techniques is that they require high annealing temperatures [20,21]. The metal-bonding and anodic-bonding methods were used to reduce the annealing temperature of the fabrication process in References [5,23]. However, anodic bonding needs an electric field during the bonding process which adds complexity to the process and the metals may defuse into the silicon which is undesirable in CMUTs fabrication. Besides that, these bonding methods are still not optimal in terms of expensive SOI wafer requirements [5,23].

Adhesive bonding is another route to low-temperature wafer bonding [27,28]. A polymer adhesive is used as the intermediate layer for bonding the wafers together. The polymer adhesive in liquid form is coated into the substrate that, in turn, covers surface roughness and minor contamination, thus avoiding the requirements of costly surface treatments and expensive SOI wafers. Another advantage associated with the polymers is that they have very high surface energy and, due to the low-temperature process, they result in low bonding stresses. So far, benzocyclobutene (BCB) and SU-8 polymers have been successfully used as an adhesive layer for CMUT fabrication [22,24,28,29]. Among all adopted methods, the lowest temperature reported in the literature for CMUT fabrication was about $250{\;}^{\circ }C$.

In this study, the aim was to produce a CMUT for underwater applications without using SOI wafers or a complex chemical mechanical polishing (CMP) process. A polymethyl methacrylate (PMMA)-based wafer-bonding technique was investigated at a lower temperature ($180{\;}^{\circ }C$). PMMA-based bonding has already been carried out in other wafer-bonding-based applications [30]. Apart from the low temperature, it is a robust, simpler, and more economical technique than the other wafer-bonding techniques, reducing fabrication steps and cost. A thermally oxidized silicon wafer forming the cavity is bonded to a second PMMA-spun wafer that also acts as the membrane of the CMUT. The process is reduced to a single lithography and a single dry-etching step, which makes it simpler as compared to the processes involved in the other fabrication approaches. The presented CMUT was specifically designed for low-frequency underwater applications. For low-frequency applications, it is difficult to achieve and maintain a thinner membrane. However, a thicker membrane and larger cavity can produce low-frequency CMUTs [31]. Therefore, in the presented CMUT, the entire thickness of the wafer was on purpose used as the membrane. Lower membrane thickness can also be achieved by either using a thinner wafer or by using the silicon nitride wafers and adopting the steps presented in References [22,28] for achieving a thinner membrane. With the silicon nitride wafers, a nanometer-level nitride membrane can be achieved at the additional cost of silicon-etching and electrode-deposition steps. Once a thin membrane is attained, cavity gaps can also be accordingly reduced to attain higher operational frequencies.

The design, fabrication, and results of the proposed CMUT are discussed in the following sections.

## 2. Design

Transducers for underwater acoustic applications are designed to operate in the 100 kHz–2 MHz frequency range [32]. The transducer, which is the subject of this study, was intended to be used in a commercially available high-data-rate acoustic modem, so the center frequency was set to 200 kHz. A highly doped high-conductivity silicon wafer of 525 μm thickness was used as the vibrating membrane. For the given membrane thickness and operating frequency, cavity radius was calculated as 2.5 mm.

**Figure 1:**
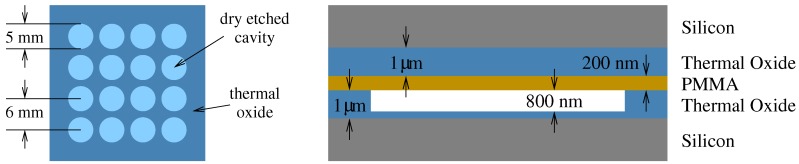
Top (**left**) and cross-sectional (**right**) views of the proposed Capacity Micromachined Ultrasonic Transducer (CMUT), showing device dimensions.

For achieving high-output pressure in transmit mode, a high cavity is required. In receive mode, on the other hand, improved sensitivity requires a small cavity gap [33]. Cavity height cannot be reduced indefinitely due to the inherent waviness of the wafer. Devices were found to be functional for a gap setting of 800 nm. The proposed technique was tested by fabricating a single-element 16-cell CMUT as shown in Figure 1.

Figure 1 shows the top view of the 16-cell CMUT along with the cross-sectional view of a single cell. The device cavity was etched into the ${\mathrm{SiO}}_{2}$ on one of the wafers, which is brought into contact with a second wafer where PMMA is used as the intermediate adhesive layer. While the high-conductivity wafers form the two electrodes of the device, they were electrically isolated by the thermal ${\mathrm{SiO}}_{2}$ layers.

The collapse voltage of the CMUT was calculated as 455 V. The parameters adopted for fabrication and analysis are summarized in Table 1.

## 3. Fabrication of CMUTs Based on PMMA Adhesive Wafer Bonding

The device was fabricated using the MEMS process steps detailed in Section 3.1, while custom-made thermocompression bonding is elaborated in Section 3.2.

### 3.1. Fabrication Process

**Figure 2:**
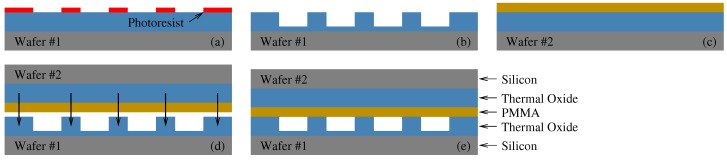
Process steps for CMUT fabrication: (**a**) pattern lithography and developing, (**b**) oxide etching and cleaning, (**c**) polymethyl methacrylate (PMMA) coating, (**d**) membrane and cavity alignment and bonding, (**e**) final structure.

Microfabrication of the devices have been carried out in Sabanci University Nanotechnology Research and Application Center (SUNUM) class 1000 clean-room facility. The process flow is depicted in Figure 2, and the steps are summarized in the subsequent paragraph.

- A 1 μm thermal oxide-deposited p-type silicon wafer was cleaned with acetone and Isopropanol and soft-baked for 20 min.
- Photoresist (MicroChemicals GmbH (Ulm, Germany) AZ5214 E) was spin-coated onto the wafer and soft-baked at $90{\;}^{\circ }C$ for 120 s. Afterward, using the photomask, the wafer was exposed to a 180 mJ$\cdot {\mathrm{cm}}^{-2}$ energy UV light using a Midas/MDA-60MS mask aligner. The sample was postbaked at $115{\;}^{\circ }C$ for 120 s and then flood-exposed at 432 mJ$\cdot {\mathrm{cm}}^{-2}$. After keeping sample for 5 min at room temperature, it was developed in AZ 726 MIF developer (MicroChem) for 75 s.
- Approximately 700–800 nm of oxide from the patterned region was etched with ${\mathrm{SF}}_{6}$ DRIE process using Oxford Plasma Lab 100 ICP300 RIE/ICP system. The sample was then cleaned with acetone and isopropanol.
- Another silicon wafer was spin-coated with 200 nm thickness 950 PMMA A4 (MicroChem). The PMMA was annealed for ≥20 min at $180{\;}^{\circ }C$ prior to the bonding to remove organic residuals and avoid bubble formation.
- Finally, both wafers were brought together and carefully aligned for bonding. The sample was wrapped in aluminum Al foil to avoid contamination. The Al foil also facilitates in uniformly distributing the pressure during the thermocompression-bonding process.

**Figure 3:**
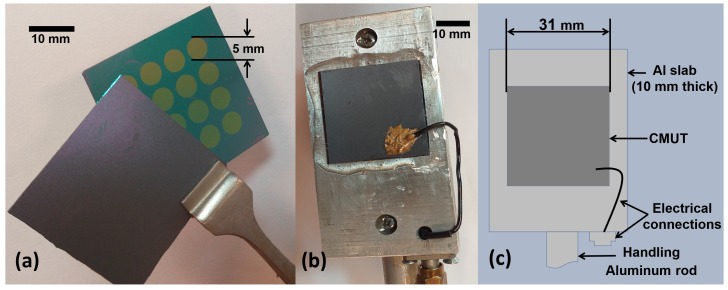
Device during fabrication stages: (**a**) before bonding, (**b**) final device, and (**c**) schematic of final device.

After successful bonding, the devices were packaged. An aluminum slab was used as a carrier that also provides electrical connectivity to one of the electrodes. A two-part epoxy was used to seal the device, and electrical connections were made through silver epoxy. Figure 3 shows the device just before bonding in the fabrication stage, and the final packaged device.

### 3.2. Thermocompression Bonding

**Figure 4:**
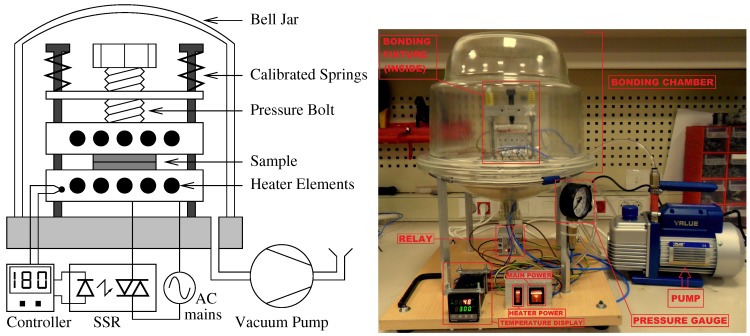
Thermocompression bonding tool: schematic (**left**), and actual (**right**).

A simple thermocompression bonder was constructed using two aluminum blocks, into each of which five 24 V 50 W ceramic heater elements were embedded (Figure 4). All heater elements were serially connected and were driven into the 220 V AC line voltage through a solid-state relay (SSR). A thermocouple was attached to one of the blocks to provide the controller with the current temperature of the heater elements. A bolted spring mechanism was used to apply constant pressure through the bonding process. Calibrated springs were used to apply 392 N (40 kgf) of force for every turn of the bolt. All the assembly was placed in a bell jar, which was evacuated by means of a low vacuum pump.

To perform the bonding, the Al-wrapped wafers were compressed in the middle of the thermal units, the bell jar was placed on the assembly, and the chamber was evacuated. Afterward, the heater elements were brought to $180{\;}^{\circ }C$. Wafers were kept under constant pressure and temperature for 60 min. Few iterations were made of gradually increasing the pressure to calculate the bonding pressure. Pressure of around 435 kPa was found to be optimal, as lower pressure values were not sufficient for bonding, and higher pressures were avoided to minimize the chances of PMMA deformation under excessive compression. After bonding, the heating was turned off and the temperature was gradually decreased to $30{\;}^{\circ }C$ before releasing the pressure and vacuum. The device was collected and prepared for testing and characterization.

## 4. Experiment Results

**Figure 5:**
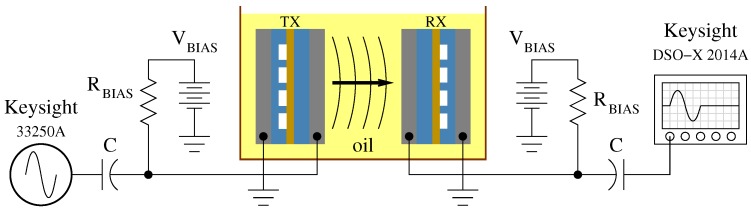
Setup for pitch–catch experiment.

Fabricated devices were tested with a pitch–catch experiment while immersed in sunflower oil. Sunflower oil was used because an insulating medium with acoustic properties close to water was needed to avoid electrical shorting of the exposed electrodes [34,35]. A pair of CMUTs, fabricated on the same wafer under identical conditions, were placed facing each other to be used as transmit and receive transducers as shown in Figure 5. Both CMUTs were biased at 136 V, and the transmitting-CMUT (TX-CMUT) was driven with a 20 $V_{\mathrm{pp}}$ two-cycle sine burst from a Keysight 33250A (Santa Clara, CA, USA) signal generator. Component values for the decoupling capacitors (C) and biasing resistors ($R_{\mathrm{BIAS}}$) were 100 nF and 100 kΩ, respectively. The received signal was recorded using a Keysight DSO-X 2014A (Santa Clara, CA, USA) oscilloscope.

### 4.1. Bandwidth

**Figure 6:**
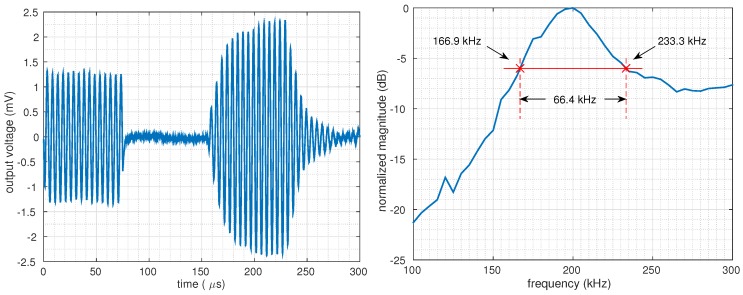
Output signal of RX-CMUT for a tone burst of 15 cycles at 200 kHz (**left**), normalized magnitude for a frequency sweep from 100 to 300 kHz (**right**).

The bandwidth of the transducer was measured by sweeping the frequency of the input signal from 100 to 300 kHz, and recording the amplitude of the received signal at every sweep point. For every measurement, a tone burst of at least 15 cycles was applied so that the steady-state output voltage could be measured. The plot on the left of Figure 6 shows the received signal for a sine burst in the time domain at 200 kHz, while the right figure shows the normalized magnitude. The center frequency was found to lie in the range of 195–200 kHz, while the full width at half maximum (FWHM) bandwidth of the device was measured as 66.4 kHz, accounting for a fractional bandwidth of 33.6%.

### 4.2. Sensitivity

Calculation of the device’s input sensitivity required knowledge of input pressure. The output pressure of the TX-CMUT was analytically found by matching the electrical measurements to a finite-element-analysis (FEA) model of the experiment setup. Details of the model, which was created in ANSYS Multiphysics (V18.1, ANSYS, Inc., Canonsburg, PA, USA), are explained in Appendix A.1.

The constructed electromechanical FEA model is capable of driving the TX-CMUT from the electrical port of the device and calculating the output of the receiving-CMUT (RX-CMUT) as electrical quantities, mimicking the experiment conditions. This model was then used to tune the device parameters to match the experiment data. Free parameters were taken as the Young’s modulus of the membrane plate and the effective gap height (as it might vary due to surface waviness). Initial simulations showed that the experiment data had significant damping. This was attributed to squeeze-film damping, which becomes significant in CMUTs with extreme radius-to-gap-height ratios (which is 2.5 mm to 800 nm for the manufactured devices). To incorporate this effect, viscous dampers were added to the FEA model, whose parameters were empirically determined to match the experiment data. Analysis showing the presence of squeeze-film damping in the fabricated devices is provided in Appendix A.2. Diffraction loss and absorption in the coupling fluid were ignored due to the extremely small distance between the TX and RX transducers and low frequency of the operation.

**Figure 7:**
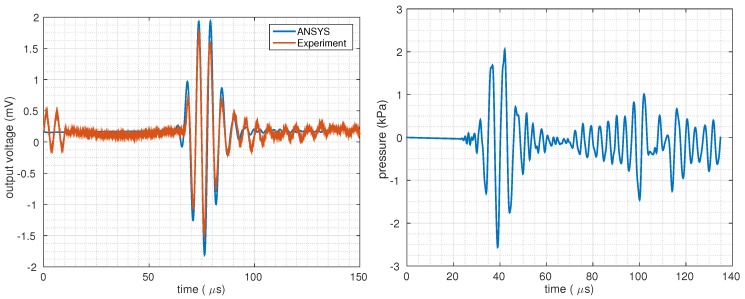
Comparison of experimental and analytical unamplified output signal (**left**), corresponding acoustic pressure (**right**).

The simulated output voltage of the RX-CMUT is superimposed on the experiment data in Figure 7. The corresponding acoustic pressure in the fluid column was then extracted from the FEA model. A 20 $V_{\mathrm{pp}}$ two-cycle sine burst at 200 kHz with 136 V DC bias was found to produce approximately 4 ${\mathrm{kPa}}_{\mathrm{pp}}$ pressure in the coupling fluid.

In response to the input pressure, RX-CMUT produces a voltage signal of 3.30 ${\mathrm{mV}}_{\mathrm{pp}}$ and hence, a sensitivity of −241.7 dB (re 1V/1μPa) without amplification was observed. The sensitivity is comparable to a reported adhesive-based wafer-bonded CMUT. The reported CMUT has a transimpedance amplifier sensitivity of −232.5 dB (re 1V/1μPa) with operational frequency of 3 MHz [22].

## 5. Discussion

### 5.1. Sensitivity vs. Bias Voltage

**Figure 8:**
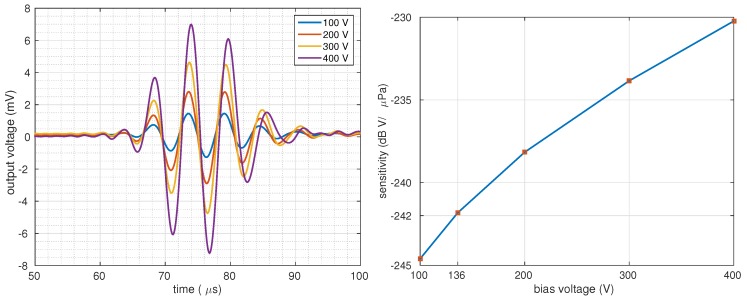
Simulated RX-CMUT output with varying bias voltage (**left**); associated sensitivity (**right**).

The PMMA-bonded CMUT, when biased at 136 V, has a receive sensitivity of −241.7 dB (re 1V/1μPa without any amplification. These results show that the sensitivity of the proposed device matches that of commercially available hydrophones such as the BII-7180 series of Benthowave Instruments Inc. [36]. CMUTs can be biased at voltages as high as 80% of their collapse voltage [37], which was calculated as 455 V for the manufactured device using the FEA model. The same model was then incorporated to calculate the sensitivity for higher bias voltages. The generated output voltages and their corresponding sensitivities are sketched in Figure 8.

Results show that the sensitivity can be increased up to −231.5 dB (re 1V/1μPa at a bias of 364 V (80% of 455 V). Observed in the figure, the resonance frequency of the device drops with an increasing DC bias due to the spring-softening effect.

### 5.2. Comparison of Fabrication Methods

The developed PMMA-based CMUTs are compared with the other published fabrication processes to assess their complexity, cost, equipment requirements, and flexibility.

System complexity is one of the comparison parameters. Wafer-bonding techniques are much simpler as compared to sacrificial-release fabrication methods, as the latter need a number of adjustments and selections such as alignment steps, choosing the proper etchant and materials for the sacrificial layer and membrane. Low-pressure chemical-vapor deposition silicon nitride (LPCVD SiN)-based wafer direct bonding needs CMP treatment. Anodic bonding needs an electric field during the bonding process that adds complexity to the process. SOI-wafer-based wafer direct bonding and adhesive bonding are considered simple, as they do not have additional bonding requirements.

Wafer cost is another comparison parameter, even though wafer cost is a very small fraction of the total when compared to clean-room equipment costs. However, the use of expensive SOI wafers in wafer-boding techniques makes wafer cost an important parameter.

The number of fabrication steps reflects equipment requirements and its usage costs. Furthermore, more fabrication steps also add to the chances of error, which depends on the efficiency of the used steps. It is therefore preferable to have fewer fabrication steps in order to avoid the mentioned problems.

Adhesive bonding techniques are flexible as compared to other methods. The other fabrication methods are very strict in the selection of wafers and their surface smoothness.

The processes were evaluated based on material cost, temperature requirement, deposition and etching steps, lithography steps, system simplicity, and special bonding restrictions. The evaluation parameters are summarized in Table 2. According to the data, PMMA-based fabrication is simple and highly cost-effective.

## 6. Conclusions

In this paper, we presented a PMMA-based wafer-bonding technique to fabricate CMUTs for underwater applications. The PMMA-based fabrication process is simpler and more cost-effective in terms of material and equipment requirements as compared to other reported wafer-bonding methods. Bonding was carried out at a low temperature in a custom-built thermocompressor. FEM analyses were carried out for CMUT characterization, and the results were compared with the experiment results. The transducer had a central frequency between 195 and 205 kHz, with a bandwidth of 66.4 kHz in immersion. When biased at 136 V, the transducer had a received sensitivity of −241.7 dB (re 1V/1μPa) without any amplification. FEA results show that sensitivity can be improved by increasing bias voltage. These results show that the presented transducer is feasible for underwater applications.

## Figures and Tables

**Table 1:** CMUT design parameters.

Parameter	Value
Resonant frequency (kHz)	200
Collapse voltage (V)	455
Bias voltage (V)	136
Membrane thickness (μm)	525
Membrane radius (mm)	2.5
Cavity height (nm)	800

**Table 2:** Comparison of fabrication methods.

Comparison Criteria	Fabrication Technique					
		Wafer Direct Bonding			Adhesive Bonding	
	Sacrificial Layer-Based [19]	SOI Wafer-Based [17,21]	Non-SOI Wafer-Based [20]	Anodic Bonding [5]	Other Methods [22,28]	* PMMA-Based
Wafer pair	N/A	SOI	LPCVD SiN	SOI	LPCVD SiN	${\mathrm{SiO}}_{2}$
		Si	LPCVD SiN	Si	Si	${\mathrm{SiO}}_{2}$
Wafer cost	low	high	low	high	low	low
Special requirements	N/A	N/A	CMP required	Electric field	BCB as adhesive	PMMA as adhesive
Surface-quality restriction	N/A	Restrict	Restrict	Restrict	low	low
Max Temp	$785{\;}^{\circ }C$	$1100{\;}^{\circ }C$	$1000{\;}^{\circ }C$	$350{\;}^{\circ }C$	$240{\;}^{\circ }C$	$180{\;}^{\circ }C$
Deposition required	yes	yes	yes	yes	yes	no *
# of lithography steps	5	4	3	3	3	1 *
# of wet etching steps	3	3	1	3	2	0 *
# of dry etching steps	4	2	2	1	2	1 *
Simplicity	low	high	high	low	high	highest

* The presented CMUT was specifically used for low frequencies. Higher frequencies can also be achieved at the cost of additional etching and metal-deposition steps as discussed earlier in Section 1.

**Figure A1:**
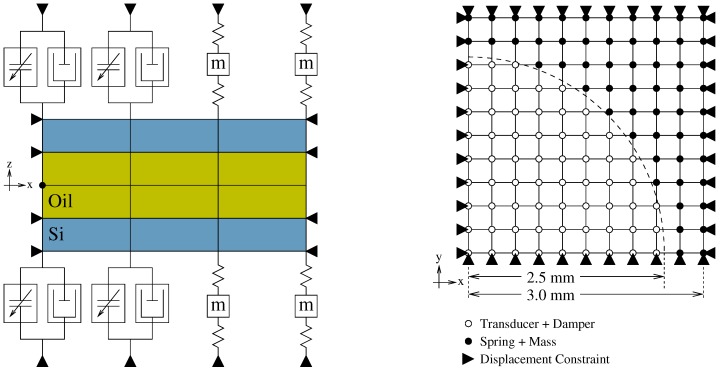
Side view (with reduced element count) and top view of FEA model.

## Appendix A

### Appendix A.1. Finite Element Analysis

The pitch–catch experiment was modeled in ANSYS Multiphysics (ANSYS, Inc., Canonsburg, PA, USA) using the mesh structure depicted in Figure A1. Only a quarter of a single transducer with symmetric boundary conditions were used in the model to reduce mesh size and simulation time. A column of oil was placed between two Si layers that acted as the plates of the transmitting and receiving transducers. FLUID30 and SOLID45 elements were used for modeling the oil and silicon layers, respectively.

The active area of the transducer is defined by placing TRANS126 Electromechanical Transducer elements to nodes that are within a circle defining the radius of the transducer, while the remaining nodes are brought to contact with the bonding PMMA layer. Owing to the extremely unmatched relative thickness of the plate and PMMA layer (while the Si layer was 525 μm, the PMMA layer was only 200 nm thick) which would make meshing impractical, the PMMA layer was modeled using a pair of springs with a central mass. The transmitter was driven from the electrical port by 136 V DC bias and a two-cycle RF-burst of amplitude 10 $V_{\mathrm{pp}}$ at 200 kHz. The squeeze-film damping effect was modeled using COMBIN14 elements, whose damping coefficients were empirically found based on experiment data. The model had 10 × 10 elements in the xy plane, while the fluid column of 10cm height was meshed using 10 elements per acoustic wavelength at 200kHz, totaling 14,428 elements, and 17,058 nodes. Table A1 lists the simulation parameters.

**Table A1:** Simulation parameters.

Parameter	Value	Units
Young’s modulus of Si	130	GPa
Poisson’s ratio of Si	0.27	
Mass density of Si	2280	$\mathrm{kg}/m^{3}$
Speed of sound in oil	1500	m/s
Mass density of oil	1000	$\mathrm{kg}/m^{3}$
Young’s modulus of PMMA	3	GPa
Mass density of PMMA	1185	$\mathrm{kg}/m^{3}$
Damping coefficient	0.06	N·s/m

### Appendix A.2. Squeeze-Film Damping

The bonding equipment used in the manufacturing of the devices was operated at low-vacuum levels, due to which the squeeze-film damping effect was observed. Linearized compressible Reynolds equation could be used to study the conduct of trapped air between two parallel vibrating plates [38]. In a solution for parallel-moving vented circular plates, Blech [39] showed that dimensionless viscous and elastic damping behavior is dependent on squeeze number (σ), given in Equation (A1).

(A1) $$\sigma =12\mu_{\mathrm{eff}}\frac{\omega }{p_{a}}{\left(\frac{a}{g_{h}}\right)}^{2}$$

where $p_{a}$ the ambient pressure, $a/g_{h}$ is the radius to cavity height ratio, ω is the angular oscillation frequency and $\mu_{\mathrm{eff}}$ is the rarefaction effect and expressed as follows.

(A2) $$\mu_{\mathrm{eff}}=\frac{\mu }{1+9.638\;k_{n}^{1.159}}$$

where μ is is the dynamic viscosity of air, and $K_{n}$ $=\lambda /g_{h}$ is the Knudsen number. The mean free path of air (λ) depends on air-pressure level (*P*) and air density (ρ) within the cavity, and is given by the following expression [40]:

(A3) $$\lambda =\frac{\mu }{u}\sqrt{\frac{\pi }{8\rho P}}$$

where u is a numerical factor equal to 0.4987445.

The parameters used in the above expressions for squeeze-number calculation and their corresponding values are summarized in Table A2.

**Table A2:** Squeeze-number computation parameters.

Parameter	Value	Units
Dynamic viscosity of air μ	$1.825\times 10^{5}$	kg/m·s
Angular frequency ω	$1.26\times 10^{6}$	rad/s
Membrane radius a	2.5	mm
Cavity height $g_{0}$	800	nm
Ambient pressure $P_{a}$	101,325	Pa
Cavity pressure P	from Table A3	Pa
Density of air ρ	from Table A3	$\mathrm{kg}/m^{3}$
Mean free path of air λ	from Table A3	nm

The squeeze number at different cavity pressure levels is calculated by inserting Expressions (A2) and (A3) into Equation (A1), as presented in Table A3.

**Table A3:** Squeeze number at different pressure levels.

Pressure (Pa)	Density ($\mathrm{kg}/m^{3}$)	Mean Free Path, λ (nm)	Squeeze Number
100,000	1.16864	67.99	17,300
10,000	0.116864	679.9	2990
1000	0.0116864	6799	231
500	0.00584	13,599	104
300	0.00351	22,665	58

Since the CMUT acts like a flexible plate instead of a rigid piston, the nonuniform deflection of the membrane results in nonuniform distribution of pressure within the cavity. Due to the pressure difference, air flows from higher-pressure regions to lower-pressure regions in the sealed cavity, which consequently results in viscous loss [41,42]. Galisultanov et al. investigated the damping effect in CMUTs and revealed that, for a squeeze number higher than 50, CMUTs possess a damping effect as, at a high squeeze number, the viscous damping force is similar for both sealed and open cavities [41,42]. The data in Table A3 show that the proposed CMUT carries a damping effect for the cavity pressure of as low as 300 Pa as per the findings of References [41,42].
